# MISNet: multi-source information-shared EEG emotion recognition network with two-stream structure

**DOI:** 10.3389/fnins.2024.1293962

**Published:** 2024-02-14

**Authors:** Ming Gong, Wei Zhong, Long Ye, Qin Zhang

**Affiliations:** ^1^Key Laboratory of Media Audio and Video (Communication University of China), Ministry of Education, Beijing, China; ^2^State Key Laboratory of Media Convergence and Communication, Communication University of China, Beijing, China

**Keywords:** EEG signals, emotion recognition, transfer learning, multi-source domain, domain adaptation

## Abstract

**Introduction:**

When constructing machine learning and deep neural networks, the domain shift problem on different subjects complicates the subject independent electroencephalography (EEG) emotion recognition. Most of the existing domain adaptation methods either treat all source domains as equivalent or train source-specific learners directly, misleading the network to acquire unreasonable transfer knowledge and thus resulting in negative transfer.

**Methods:**

This paper incorporates the individual difference and group commonality of distinct domains and proposes a multi-source information-shared network (MISNet) to enhance the performance of subject independent EEG emotion recognition models. The network stability is enhanced by employing a two-stream training structure with loop iteration strategy to alleviate outlier sources confusing the model. Additionally, we design two auxiliary loss functions for aligning the marginal distributions of domain-specific and domain shared features, and then optimize the convergence process by constraining gradient penalty on these auxiliary loss functions. Furthermore, the pre-training strategy is also proposed to ensure that the initial mapping of shared encoder contains sufficient emotional information.

**Results:**

We evaluate the proposed MISNet to ascertain the impact of several hyper-parameters on the domain adaptation capability of network. The ablation experiments are conducted on two publically accessible datasets SEED and SEED-IV to assess the effectiveness of each loss function.

**Discussion:**

The experimental results demonstrate that by disentangling private and shared emotional characteristics from differential entropy features of EEG signals, the proposed MISNet can gain robust subject independent performance and strong domain adaptability.

## 1 Introduction

Emotion is critical in influencing people's decision-making, social interaction and evaluation of things (Dolan, 2002). By incorporating emotional analysis into human-machine interactions, the machines can better understand humanity and become more natural (Picard, 2001). Numerous studies have been conducted on emotion recognition based on various modes, such as facial expressions (Ko, 2018), speech (Schuller, 2018) and electrophysiological signals. Electroencephalography (EEG) stands out among these signals due to its objective properties and high temporal resolution benefits (Yang et al., 2021). Specifically, EEG-based affective brain-computer-interfaces (aBCIs) (Mühl et al., 2014) aim to detect affective states from EEG signals and use them in various applications, such as estimating driver drowsiness to improve driving safety (Wu et al., 2016; Cui et al., 2019; Jiang et al., 2020) and establishing an objective detection system for depression (Cai et al., 2020) or post-traumatic stress disorder (Rozgic et al., 2014) to enable self-diagnosis.

Regarding EEG emotion recognition, depending on the head size, body state and experimental environment, the structural and functional variability of the brain may vary between the subjects, resulting in substantial differences in the collected EEG signals (Samek et al., 2013). Traditional machine-learning algorithms usually train a classifier such as support vector machines (Zheng and Lu, 2016) or random forests (Gupta et al., 2018), by utilizing data from a limited number of objects. Nevertheless, due to the EEG signals do not satisfy the independent and identically distributed condition which is caused by the individual difference and non-stationary properties, directly using the subject-dependent models to detect the emotional states of a new subject decreases recognition accuracy. Although collecting a large amount of labeled data from the new subject and using them to fine-tune the classifier is an obvious solution, it is time-consuming and degrades the subjects' experience significantly (Zhao et al., 2021). Hence, this strategy cannot be utilized in the practical aBCI applications.

The unsupervised domain adaptation is an alternative method to align different distribution domains, bridging the existing labeled subjects and new unlabeled ones by identifying their similarities (Wang and Chen, 2021). However, without access to the target domain, it is challenging to train a well-generalized network (Blanchard et al., 2011; Zhou et al., 2023). In contrast, the performance of unsupervised domain adaptation approaches is typically enhanced in the training phase by using unlabeled data from the target domain and employing instance-based, model-based, or feature-based (Wang and Chen, 2021) methods.

Compared with traditional machine learning, using deep learning to solve domain adaptation problems has relatively low requirements for trainers to select features. Based on the significant advancements in computer vision, speech recognition and natural language processing, we believe that the deep learning methods have potential in EEG emotion recognition. Regarding EEG emotion recognition, there have been sufficient studies on subject-dependent experiments (Kim and André, 2008; Ding et al., 2020; Nath et al., 2020; Pan et al., 2021; Zhang et al., 2022; Song et al., 2023). Several experimental results indicate that deep learning has a great potential for solving domain adaptation problems (Craik et al., 2019; Roy et al., 2019). When using deep learning in aBCI domain adaptation applications, most works regarded all source domains as being the same (Li H. et al., 2018; Li Y. et al., 2018; Luo et al., 2018). Hence all source domains should be merged into the common domain to extract features. This strategy disregards the distribution difference inside the source domains, resulting in the model being unable to train to the optimal effect. When there are outlier source domains, the model is difficult to converge, leading to “negative transfer”. On the other hand, some researchers identified the distribution difference mentioned above and trained domain-specific networks directly (Chen et al., 2021a,b; Luo and Lu, 2021), marginally improving recognition performance but overlooking the commonality among source domains. Furthermore, most of these approaches need to judge the distance between the features of target domain and those of each source domain to select one or several similar source domains, and weight the predictions to form the final prediction. When one source domain has larger distance to others, which means there is an outlier source domain, it may occur that one private domain mapping of target domain is far from other private domain mappings, and the model performance would decrease. Therefore, it is necessary to consider the individual difference and group commonality among the multi-source domains, further improving recognition performance.

This paper considers the individual difference and group commonality of multi-source domains and proposes the multi-source information-shared EEG emotion recognition network based on marginal distribution. In the proposed network, the domain-specific and domain-shared features are extracted and combined dynamically to alleviate the negative transfer problem. Specifically, we first integrate a pre-training strategy into the network to maximize the utilization of current source domain data and reasonably initialize the network, further enhancing its stability. Then, we extract domain-specific features by using private encoders and domain-shared features by a pre-trained shared encoder to represent the individuality and commonality of EEG signals from different domains. Besides employing the maximum mean discrepancy to align the marginal distributions between the source and target domains, two auxiliary loss functions are also designed to improve the astringency of network and align the distributions of private target domains. These loss functions can further enhance the mapping capability of private encoders by considering the information of other private domains. Moreover, rather than heuristically altering the weights of classifiers, we integrate the outputs of classifiers according to the domain-specific and domain-shared feature distributions, thereby dynamically optimizing the network. The experimental results on the SEED and SEED-IV datasets validate the performance of proposed method.

The main contributions of this paper are summarized as follows:

- We propose an efficient EEG emotional recognition network that incorporates the individual difference and group commonality of multi-source domains.
- We design a two-stream training structure and loop iteration strategy to compute two auxiliary loss functions *L*_*was*−*gp*_ and *L*_*diff*−*gp*_ for aligning the marginal distributions of domain-specific and domain-shared features in target domains. Furthermore, the gradient penalty is also constrained on the above two losses to improve the stability of network.
- We introduce the subject-dependent pre-training process to initialize the shared encoder with reasonable parameters, which supplies emotional information to the shared domain.

The remainder of this paper is organized as follows. Section 2 introduces the related works on domain adaptation and EEG-based subject independent emotion recognition. Section 3 proposes the multi-source information-shared network and illustrates the corresponding training process. Section 4 presents the experimental settings and implementation details. Subsequently in Section 5, the results of the ablation experiments are analyzed and the comparisons are made on the SEED and SEED-IV datasets. Finally, Section 6 concludes this work and suggests future research directions.

## 2 Related work

This section briefly reviews the concept and methods of domain adaptation and then introduces the relevant work on EEG-based subject independent emotion recognition.

### 2.1 Domain adaptation

In domain adaptation, which is a rapidly growing transfer learning direction, the labeled source and unlabeled target domains share the same features and categories. The domain adaptation focuses on using the source domain knowledge to process the target domain features when the source and target domain distributions are different (Wang and Chen, 2021). Adopting deep learning for domain adaptation can automatically extract more expressive features and meet the end-to-end needs of practical applications. Typically, three categories are available: instance-based learning, model-based learning, and feature-based learning. The instance-based learning aims to select and weight samples from the source and target domains (Blitzer et al., 2006; Li et al., 2016). The objective of model-based learning is to transfer parameters between different models. By mapping the different probability distributions of the source and target domains, the feature-based learning characterizes the similarity between the source and target domains, which can be classified as marginal, conditional, joint or dynamic distribution adaptation.

In many practical applications involving multiple source domains, the multi-source domain adaptation methods can be used to transfer knowledge from multiple domains and consider domain shifts among source domains to achieve better transfer results. Recently, Zhao et al. (2018) bridged deep learning and multi-source domain adaptation by developing a multiple-domain discriminator to align the features of source and target domains, which is a typical adversarial discriminative method. Xu et al. (2018) constructed multiple domain discriminators and classifiers for each source-target domain pair. Then the target labels are voted according to the distribution-weight combining rule. Zhu et al. (2019) extracted distinct source domains into distinct feature spaces and aligned the source and target domains across each feature space. Moreover, they reduced the variance of the classifier output through consistency regularization to directly average the output of classifier and avoid the artificial setting.

### 2.2 EEG-based subject independent emotion recognition

Since the differences in gender, body state and experimental environment between individuals will lead to different neurophysiological activity patterns, the EEG signals of different subjects do not satisfy the independent and identically distributed condition. In this scenario, the issue of domain shift has arisen, that is, under the same emotional stimulus, different individuals may have different EEG responses, resulting in inconsistent distribution of collected EEG signals. The domain shift problem is the main challenge that the subject-independent algorithms need to address. It not only appears in the different sources of EEG data, but also may appear in the same EEG source due to psychological changes of the participants or technical factors, which greatly limiting the performance of the model.

To reduce inter-subject variability, transfer learning in EEG emotion recognition has two primary branches: domain adaptation and domain generalization. Through the data manipulation, representation learning and learning strategy, the domain generalization aims to learn a model from multiple source domains that generalizes to unseen target domains (Wang et al., 2023). Since the domain generalization methods do not utilize the information of target domain during training, they rarely obtain high recognition accuracy. In contrast, the domain adaptation methods use the information from target domain to transfer knowledge while minimizing domain shifts between the source and target domains. Zheng and Lu (2016) applied transfer component analysis (TCA) and transductive parameter transfer (TPT) to the subject independent EEG emotion recognition on the SEED dataset. Li H. et al. (2018) suggested an alternative method by employing the domain-adversarial neural network (DANN), which involves the adversarial training of feature encoder and domain classifier. Luo et al. (2018) proposed the wasserstein GAN domain adaptation network (WGANDA) by using the gradient penalty to alleviate the domain shift problem. By considering multi-source domain adaptation, Luo and Lu (2021) proposed the wasserstein-distance-based multi-source adversarial domain adaptation (wMADA), which regarded different subjects as different domains and designed an adaptive weight strategy considering the relationship between each domain. Zhao et al. (2021) developed a plug-and-play domain adaptation (PPDA) network, which disentangles the emotional information by considering the domain-specific and domain-invariant information simultaneously. Chen et al. (2021b) took the source data with different marginal distributions into account and proposed a multi-source EEG-based emotion recognition network (MEERNet). Later, they used the disc-loss to improve domain adaptation ability and proposed another multi-source marginal distribution adaptation (MS-MDA) network for subject independent and cross-session EEG emotion recognition (Chen et al., 2021a).

It should be noticed that, most of the existing domain adaptation methods mentioned above either treat all source domains as equivalent or train source-specific learners directly, misleading the network to acquire unreasonable transfer knowledge and thus resulting in negative transfer. Therefore, this paper considers the feature individuality and commonality of distinct domains and weights similar domains based on their feature distributions, enhancing recognition performance.

## 3 Methods

In this section, we present the entire architecture and its data transmission process. And then the involved modules are analyzed in detail, and the loss functions are also designed by aligning different domains.

### 3.1 Framework

**Figure 1:**
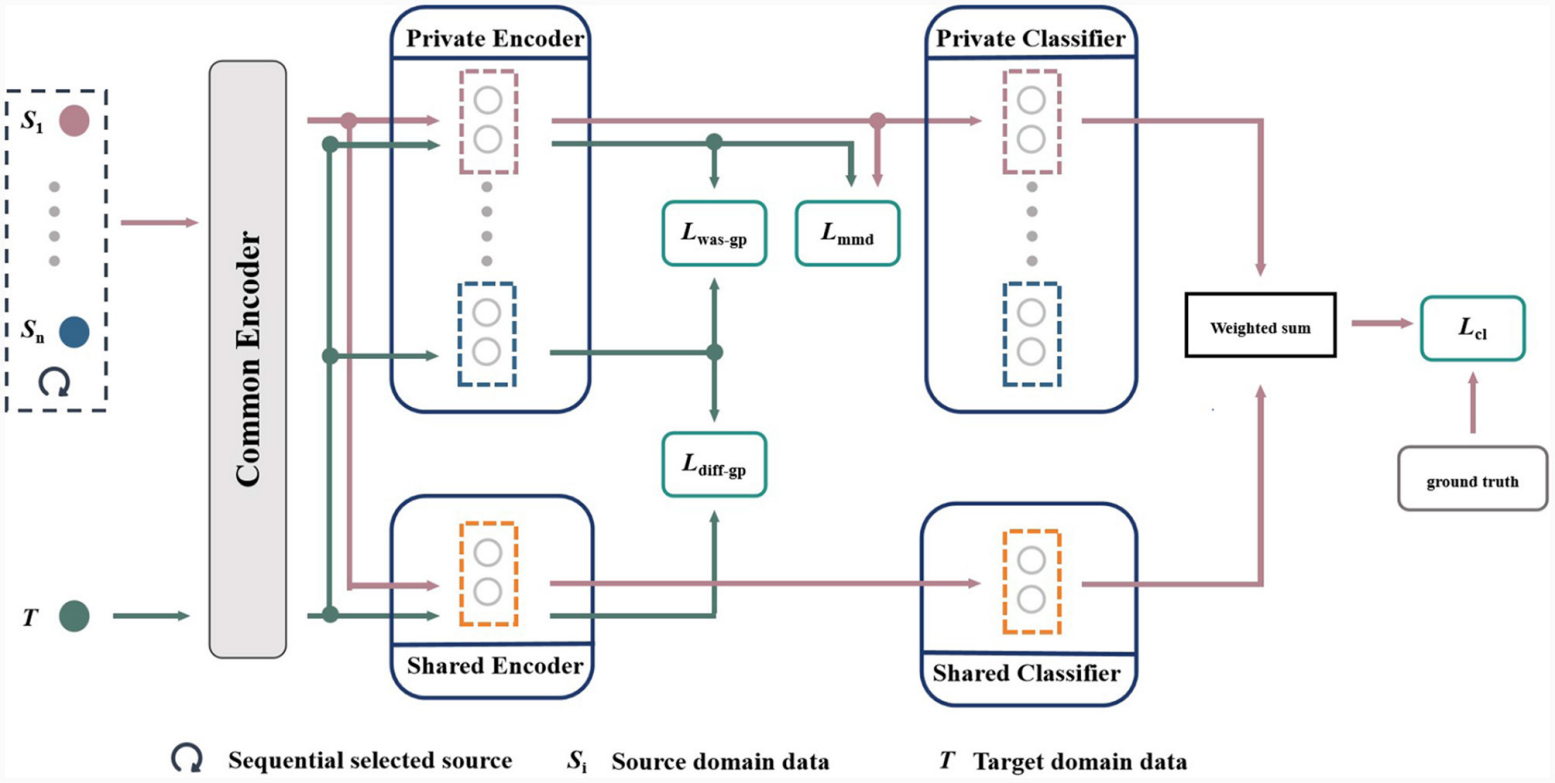
Overall framework of the proposed MISNet network.

The main challenge we aim to address is the domain shift problem caused by the non-stationary of EEG signals and the individual differences among users. Figure 1 shows the framework of the proposed multi-source information-shared network (MISNet) based on marginal distribution, which comprises five components: common encoder, private encoders, shared encoder, private classifiers and shared classifier. In Figure 1, the pink lines, shapes and arrows represent the path of the EEG data matrix **X**_*S*_ from the source domains, and the green lines, shapes and arrows represent that of the EEG data matrix **X**_*T*_ from the target domain. For each domain, the low-level features are firstly extracted by the common encoder, and then the private encoders are constructed to extract domain-specific information, while the shared encoder extracts the domain-independent information. Subsequently, four loss functions in green squares stand for *L*_*mmd*_, *L*_*was*−*gp*_, *L*_*diff*−*gp*_ and *L*_*cl*_, which are analyzed in detail in Section 3.2. Finally, the predictions from private classifiers and shared classifier are weighted and summed by the similarity between the source and target features.

Specifically, we sequentially select one subject in the dataset as the target domain data, and the other subjects as the source domain data. As described in Equation (1), let **X**_*S*_ be the source data matrices, **Y**_*S*_ are their labels, and **X**_*T*_ are the unlabeled target data matrices,


(1) $$\begin{array}{l} X_{S}={\left\{X_{S}^{i}\right\}}_{i=1}^{n},Y_{S}={\left\{Y_{S}^{i}\right\}}_{i=1}^{n}, \end{array}$$

where *n* represents the number of subjects in source domains.

In the proposed MISNet, the common encoder *E*_*C*_ maps the source data matrices ${\text{X}}_{S}^{i}$ and target data matrices **X**_*T*_ to the low-level feature space as shown in Equation (2),


(2) $$\begin{array}{l} {X_{S}^{\prime }}^{i}=E_{C}\left(X_{S}^{i}\right),X_{T}^{\prime }=E_{C}\left(X_{T}\right). \end{array}$$

Then for the low-level features of each source domain, we construct a private encoder to obtain its source domain-specific characteristics. For the target domain, the *n* private encoders extract domain-specific features as shown in Equation (3),


(3) $$\begin{array}{l} F_{SP}^{i}=E_{P}^{i}\left({X_{S}^{\prime }}^{i}\right),F_{TP}^{i}=E_{P}^{i}\left(X_{T}^{\prime }\right),i=1,2,\ldots ,n, \end{array}$$

where *n* is the number of source domains. Meanwhile as shown in Equation (4), the shared encoder *E*_*S*_ maps the low-level features of source and target domains to the shared domain,


(4) $$\begin{array}{l} F_{SS}^{i}=E_{S}\left({X_{S}^{\prime }}^{i}\right),F_{TS}=E_{S}\left(X_{T}^{\prime }\right). \end{array}$$

Subsequently as described in Equation (5), the private classifier $C_{P}^{i}$ and shared classifier *C*_*S*_ take (${\text{F}}_{SP}^{i},{\text{F}}_{TP}^{i}$) and (${\text{F}}_{SS}^{i},{\text{F}}_{TS}$) as the inputs and output the emotion predictions (${\hat{\text{Y}}}_{SP}^{i},{\hat{\text{Y}}}_{TP}^{i}$) and (${\hat{\text{Y}}}_{SS}^{i},{\hat{\text{Y}}}_{TS}$), respectively,


(5) $$\begin{array}{l} \begin{array}{c} {\hat{Y}}_{SP}^{i}=C_{P}^{i}\left(F_{SP}^{i}\right),{\hat{Y}}_{TP}^{i}=C_{P}^{i}\left(F_{TP}^{i}\right),\\ {\hat{Y}}_{SS}^{i}=C_{S}\left(F_{SS}^{i}\right),{\hat{Y}}_{TS}=C_{S}\left(F_{TS}\right). \end{array} \end{array}$$

Finally, ${\hat{\text{Y}}}_{S}$ is the weighted sum of ${\hat{\text{Y}}}_{SP}^{i}$ and ${\hat{\text{Y}}}_{SS}^{i}$, ${\hat{\text{Y}}}_{T}$ is that of ${\left\{{\hat{\text{Y}}}_{TP}^{i}\right\}}_{i=1}^{n}$ and ${\hat{\text{Y}}}_{TS}$, respectively. We use ${\hat{\text{Y}}}_{S}$ to calculate the classification loss *L*_*cl*_ in the training phase and ${\hat{\text{Y}}}_{T}$ to predict the emotion category in the test phase.

### 3.2 Modules

For the domain shift problem in subject independent EEG emotion recognition, we propose to design an EEG emotion recognition model based on feature disentanglement, extracting domain-specific and domain-shared features to improve the robustness and interpretability of the network. A common encoder is used to extract the low-level features of the EEG signal, and the private encoders map the sample data of each domain to its domain-specific features, reducing the distance between the source and target domains after their feature mapping. The shared encoder extracts domain-shared features and imposes secondary constraints on the mapping distance between the source domain and target domain features. The private classifiers and shared classifier map the domain-specific and domain-shared features to predict the emotions of EEG signals. This section will specifically explain the role of each module in the proposed model and the overall optimization strategy.

#### 3.2.1 Common encoder

Despite the individual differences in EEG signals, some common EEG characteristics still exist in the signals of human brain activity. We assume that EEG signals from different subjects share the same shallow feature. Similar to MS-MDA (Chen et al., 2021a) and MEER-Net (Chen et al., 2021b), a common encoder maps all domain data into a common latent space, extracting the low-level features of source and target domains. The common encoder is designed to perform the nonlinear mapping of DE features of EEG signals, which obtains the preliminary mapping of emotional information by extracting low-level features from EEG signals. This lays a solid foundation for extracting the individual difference and group commonality of multi-source domains, therefore enhancing the classification performance.

#### 3.2.2 Private encoders and shared encoder

To capture the domain-specific information and consider the difference among different domains, we set up *n* fully-connected layers as private encoder for each source domain to map the data from the common feature space to the latent private feature space. Inspired by the idea of feature disentanglement in domain generalization (Wang and Chen, 2021), the shared emotional information is extracted through the shared encoder by mapping the low-level feature space to the shared feature space. Note that the shared encoder has the same structure as the private encoder in order to balance their learning abilities. For one iteration in each epoch, the private and shared encoders capture only the features of source domain and target domain that are currently trained. We employ the maximum mean discrepancy (MMD) to calculate the marginal distribution between the source and target domains in reproducing the kernel hilbert space H. MMD is often used to measure the distance between two distributions and is a commonly used loss function in transfer learning. The definition of MMD is


(6) $$MMD(f,p,q)=\underset{\|f{\|}_{H}\leq 1}{\sup}E_{p}[f(x)]-E_{q}[f(y)],$$

where *f* is the mapping function which is the norm in the reproducing kernel hilbert space. The distributions of *x* and *y* is *p* and *q*, respectively, and *E* is the mathematical expectation. However, this equation is challenging to calculate because the feature space of *f* has infinite dimensions. Thus, Equation 6 is solved by using the linear kernel function to simplify calculation,


(7) $$\begin{array}{l} L_{mmd}=\;|\psi (F_{SP}^{i})-\psi (F_{TP}^{i}){|}_{H}\\ \;=\bar{(F_{SP}^{i}-F_{TP}^{i})*{\left(F_{SP}^{i}-F_{TP}^{i}\right)}^{T}}, \end{array}$$

where ψ denotes the mapping function, the symbol * is the matrix multiplication, and $\bar{x}$ represents the mean of *x* in feature dimension. *L*_*mmd*_ dominates the domain adaptation direction, alleviating the feature distribution difference between the source and target domains.

**Figure 2:**
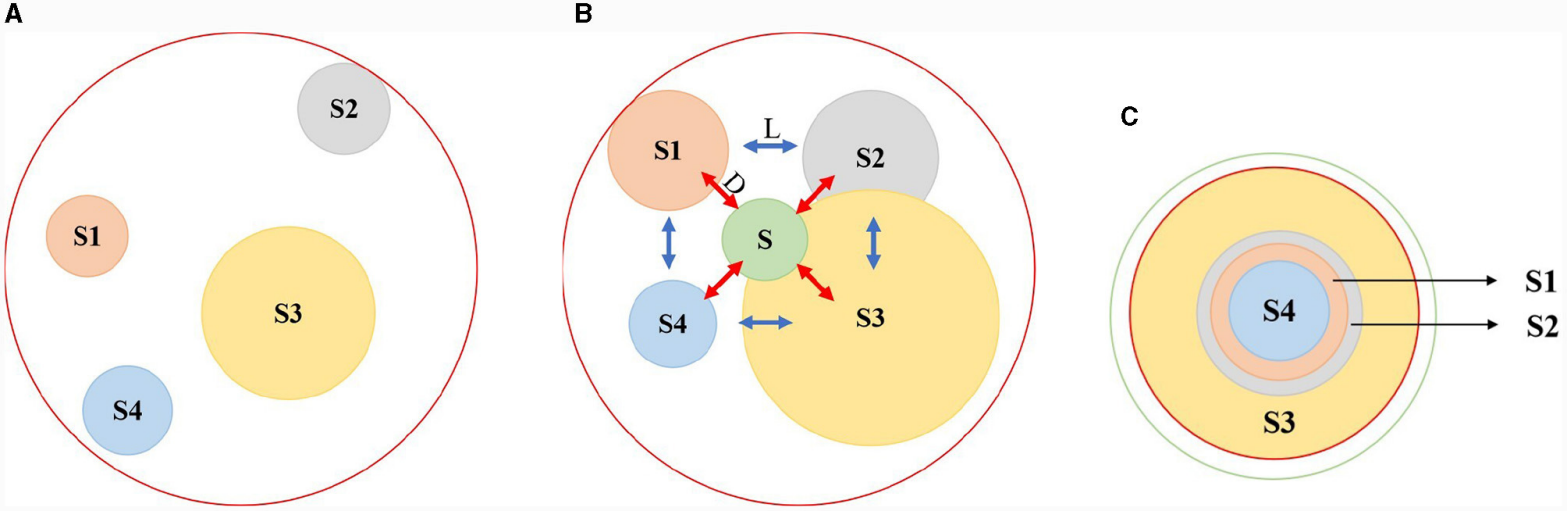
The optimization process of *L*_*was*−*gp*_ and *L*_*diff*−*gp*_. S1, S2, S3, and S4 represent the source private domains, respectively, and S is the shared domain. *L* denotes the center distance between source private domains, and *D* is the center distance between each source private domain and shared domain. The red circle symbolizes the boundary of maximum private domain, and the green circle represents the fluctuation boundary of the shared domain. **(A)** the initial states of source private domains, **(B)** the objective of optimization, and **(C)** the circumstance of optimal convergence.

Due to individual differences, all source domains are linearly independent, indicating that their private feature distributions may be quite distinct. This results in a larger spacing among all source private domains, forming a larger outer contour boundary denoted by the red circle, as shown in Figure 2A. In the process of optimizing iteration, in addition to reducing the distribution distance between the source private and target private domains, it is also necessary to shrink the spacing among source private domains, thereby obtaining a more compact set of source private domains. And thus, the overall boundary of source private domains is also reduced, denoted by the red circle as shown in Figure 2B. On the other hand in Figure 2B, the distribution distance between the shared and private domains is also reduced, forcing the network to extract the domain-independent features. In order to improve the training speed and reduce the network complexity, the above operations are not performed on the distribution distance of different domains, but on the center of each domain.

Specifically, to align the private domains and shared domain, we design two auxiliary loss functions *L*_*was*−*gp*_ and *L*_*diff*−*gp*_. In the current *i*-th iteration, the first order *L*_*was*_ is proposed to align the marginal distributions of each private domain as shown in Equation (8),


(8) $$\begin{array}{l} L_{was}=\overset{n}{\underset{j=1,j\neq i}{\sum }}|\bar{F_{TP}^{i}}-\bar{F_{TP}^{j}}|, \end{array}$$

where $\bar{{\text{F}}_{TP}^{i}}$ and $\bar{{\text{F}}_{TP}^{j}}$ denote the mean vector across feature dimensions of the domain-specific features extracted by the *i*-th and *j*-th private encoders, respectively. Considering the individual differences and potential outliers of the source domains, we select the features of the target private domain to compress the different private domains through forcing the private encoders to extract the domain-specific information from the target domain rather than the source domains. Furthermore, the soft version of the constraint with a penalty *L*_*was*−*gp*_ is enforced on the gradient norm of random samples ${\text{x}}_{T}^{i}\in {\text{X}}_{T}$ to improve the stability of *L*_*was*_ and reduce optimization errors caused by the outlier gradients,


(9) $$\begin{array}{l} L_{was-gp}=\sum_{j=1,j\neq i}^{n}\underset{L_{was}}{\underset{︸}{\left|\;\bar{F_{TP}^{i}}-\bar{F_{TP}^{j}}\right|}}+\\ \;\underset{gradient\;penalty}{\underset{︸}{\bar{{\left(\|{▽}_{{\hat{X}}_{w}}E_{P}^{i}({\hat{X}}_{w}){\|}_{2}-1\right)}^{2}}}}, \end{array}$$

where ${\hat{\text{X}}}_{w}$ is uniformly defined along straight lines between pairs of points sampled from the *i*-th target domain-specific feature ${\text{F}}_{TP}^{i}$ and *j*-th target domain-specific feature ${\text{F}}_{TP}^{j}$. This idea is motivated by the WGAN-GP (Gulrajani et al., 2017), where the gradient penalty also adopts the no-batch normalization and two-sided penalty strategy. Different from WGAN-GP, ${\text{F}}_{TP}^{i}$ and ${\text{F}}_{TP}^{j}$ are extracted from different private encoders that have the same input feature ${\text{X}}_{T}^{\prime }$. In addition, we also propose *L*_*diff*−*gp*_ to align the distributions of *i*-th private encoder and the shared encoder,


(10) $$L_{diff-gp}=\underset{L_{diff}}{\underset{︸}{\left|\bar{F_{TP}^{i}}-\bar{F_{TS}}\right|}}+\underset{gradient\;penalty}{\underset{︸}{\bar{{\left(\|{▽}_{{\hat{X}}_{d}}E_{P}^{i}({\hat{X}}_{d}){\|}_{2}-1\right)}^{2}}}},$$

where ${\hat{\text{X}}}_{d}$ is calculated similarly as ${\hat{\text{X}}}_{w}$ in Equation (9) by using the target private feature ${\text{F}}_{TP}^{i}$ and the target shared feature **F**_*TS*_ in the *i*-th iteration.

With the progress of loop iteration, the overall boundary of source private domains will shrink rapidly under the constraints of *L*_*was*−*gp*_ and *L*_*diff*−*gp*_. Given an optimal convergence as illustrated in Figure 2C, the centers of the private and shared domains approach one another. The optimal overall private domain has the smallest boundary, which is equal to the boundary of maximum private domain denoted by the red circle as shown in Figure 2C. Moreover, the spacing among the optimal private domains is also minimized or even disappeared. Meanwhile, the fluctuation boundary of the shared domain will approach the boundary of maximum private domain represented by the green and red circles as illustrated in Figure 2C. Since *L*_*cl*_ and *L*_*mmd*_ dominate the classification and domain adaptation tasks, respectively, the fluctuation boundary of the shared domain cannot easily converge to the optimum result. To sum up, the final convergence of the shared domain center meets the following three requirements:

- Meet the minimum *L*_*cl*_ requirements for the shared domain.
- After the private encoder mapping process, the source and target domains must have the minimum *L*_*mmd*_.
- Meet the minimum distance requirement between the fluctuation boundary of shared domain and the optimum boundary of private domains.

Furthermore, when there is a conflict during the optimization of the shared domain distribution and *L*_*cl*_ or *L*_*mmd*_, *L*_*cl*_ and *L*_*mmd*_ will prioritize to optimize, resulting a small spacing between the boundary of maximum private domain and that of shared domain. Under ideal circumstances, the boundary of the shared domain can be optimal, that is, being the boundary of maximum private domain. At this point, the extracted domain-independent features are optimum for emotion prediction, represented by the overlap of green and red circles in Figure 2C.

#### 3.2.3 Private and shared classifiers

Following the private encoders, the private classifiers predict emotion states by using the private features. The softmax activate function is implemented after the fully-connected layer corresponding to each source domain, which transforms hidden states to predict the category label. Like the private classifiers, the shared classifier has the same structure to balance their classification abilities. During the training process, we measure *L*_*cl*_ of private and shared classifiers using the label smoothing cross-entropy loss as described in Equations (11) and (12),


(11) $$\begin{array}{l} \begin{array}{c} L_{cl_{P}}=-\overset{K}{\underset{c}{\sum }}q\left(y,c\right)\log P\left(c|{\hat{Y}}_{SP}^{i}\right),\\ L_{cl_{S}}=-\overset{K}{\underset{c}{\sum }}q\left(y,c\right)\log P\left(c|{\hat{Y}}_{SS}\right), \end{array} \end{array}$$

(12) $$q(y,c)=\left\{ \begin{array}{l} 1-\varepsilon \;if\;c=Y_{S},\\ \varepsilon /K-1\;otherwise, \end{array}\right.$$

where *Y*_*S*_ is the emotion label of the source domain, ε is the smooth probability, and *K* is the category number of emotions.

#### 3.2.4 Weight sum

Considering both the individual differences and group commonalities, we also propose to weight *L*_*c*_*l*__*P*__ and *L*_*c*_*l*__*S*__ based on the similarity between the private target domain and private source domain to dynamically adjust the optimization process and balance the weight of the private and shared networks. During the training process, we integrate the private and shared classifiers by calculating MMD between the private and shared domains. The weight of private features *w*_*p*_ and shared features *w*_*s*_ is calculated by


(13) $$\begin{array}{l} w_{p}=|\psi \left(F_{SP}^{i}\right)-\psi \left(F_{TP}^{i}\right){|}_{H}, \end{array}$$

(14) $$\begin{array}{l} w_{s}=|\psi \left(F_{SS}\right)-\psi \left(F_{TS}\right){|}_{H}. \end{array}$$

And *L*_*cl*_ is calculated by,


(15) $$\begin{array}{l} L_{cl}=\frac{w_{s}}{w_{p}+w_{s}}*L_{cl_{P}}+\frac{w_{p}}{w_{p}+w_{s}}*L_{cl_{S}}. \end{array}$$

The weighted sum minimizes the distance between the private and shared domains. The deeper reason is the dynamic adjustment of the optimization process, as the smaller the MMD between the source and target domains, the smaller the difference in their distributions. Specifically, if the distribution between source and private shared domains are closer, *w*_*s*_ will be smaller, and then the weight of private encoder is also smaller. Due to the back propagation theory, the less gradient is assigned to private encoder, the more would be assigned to shared one relatively, indicating that the shared encoder has more learning capabilities than private encoder. Therefore, more learning capabilities are assigned to the corresponding encoder. By using Equation (15), the outputs of network are weighted based on their distributions, and thus more attentions are paid to the inter-domain predictions with more similarities.

Given *L*_*cl*_, *L*_*mmd*_, *L*_*was*−*gp*_ and *L*_*diff*−*gp*_, the final loss function is represented as,


(16) $$\begin{array}{l} L=L_{cl}+\alpha L_{mmd}+\beta L_{was-gp}+\gamma L_{diff-gp}, \end{array}$$

where α, β, and γ are the hyper-parameters. To sum up, *L*_*cl*_ is the classification loss function, which controls the overall optimization direction of the model. *L*_*mmd*_ is the domain adaptation loss function, which controls the domain adaptation direction of the model. *L*_*was*−*gp*_ aligns the marginal distributions of each private domain. *L*_*diff*−*gp*_ aligns the distributions of *i*-th private encoder and the shared encoder. By combing those four loss functions as in Equation (16), the model can obtain the ability of alleviating negative transfer by considering the individual difference and group commonality simultaneously.

In the test phase, we assume that the optimal convergence boundary has been reached, and the predictions of the private and shared domains are added together to output the final results,


(17) $$\begin{array}{l} {Ŷ}_{T}=\frac{1}{n}\overset{n}{\underset{i=1}{\sum }}\left(C_{P}^{i}\left(F_{TP}^{i}\right)+C_{S}\left(F_{TS}\right)\right). \end{array}$$

In summary, the workflow of the proposed MISNet is presented in Algorithm 1.

**Algorithm 1:**
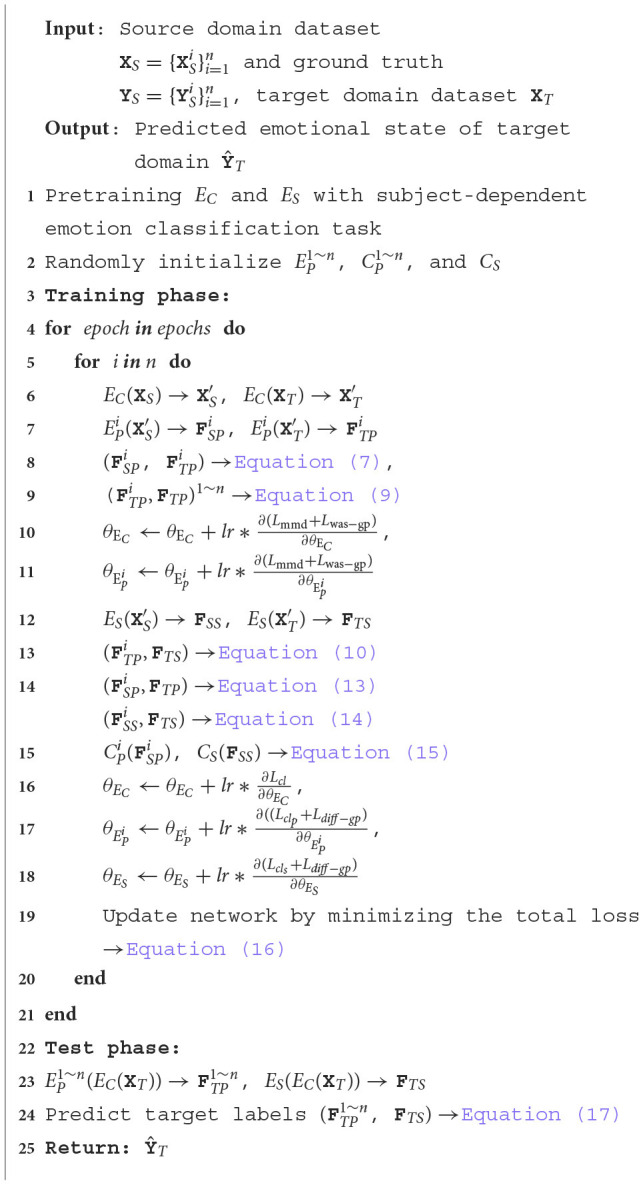
Workflow of the proposed MISNet framework.

### 3.3 Training strategy

Since the main convergence direction of different private encoders is determined by the classification loss *L*_*cl*_ of source domain and their initial feature mapping rules are determined by the distributions of source domains, when there is an outlier source domain, it may occur that one private domain mapping of target domain is far from other private domain mappings. Consider an extreme case of *L*_*was*−*gp*_ in Equation (9), when a private feature ${\text{F}}_{TP}^{i}$ is far from other private features ${\text{F}}_{TP}^{j}$ (*j*≠*i*) in the reproducing kernel hilbert space. If directly using the traditional parallel training strategy, this outlier will affect other private encoders and classifiers due to the addition operation of loss function. Therefore, the prediction and mapping rules of private encoder in the outlier source domain is significantly different from other private encoders, resulting in a large difference between ${\text{F}}_{TP}^{i}$ and ${\text{F}}_{TP}^{j}$, ultimately impacting the training process.

In the above case, *L*_*diff*−*gp*_ in Equation (10) will also be somewhat affected. Since one or more source domains may be distant from other sources, the mapping rules will have large differences even when inputting the same low-level feature of the target domain. Additionally, the model training deviation caused by the outlier source domains is mixed in the shared domain, misguiding the optimization direction of shared encoder and the convergence boundary of shared classifier. The distance between the private domain mapping of outlier source and the shared domain will inevitably cause the outlier domain mapping to stay away from other source domain mappings. In this case of improper optimization, the model tends to converge to most source domains while ignoring the outlier domain, the boundary of shared domain shifts toward the concentrated source domains and deviates from that of maximum private domain, resulting the model not to converge.

**Figure 3:**
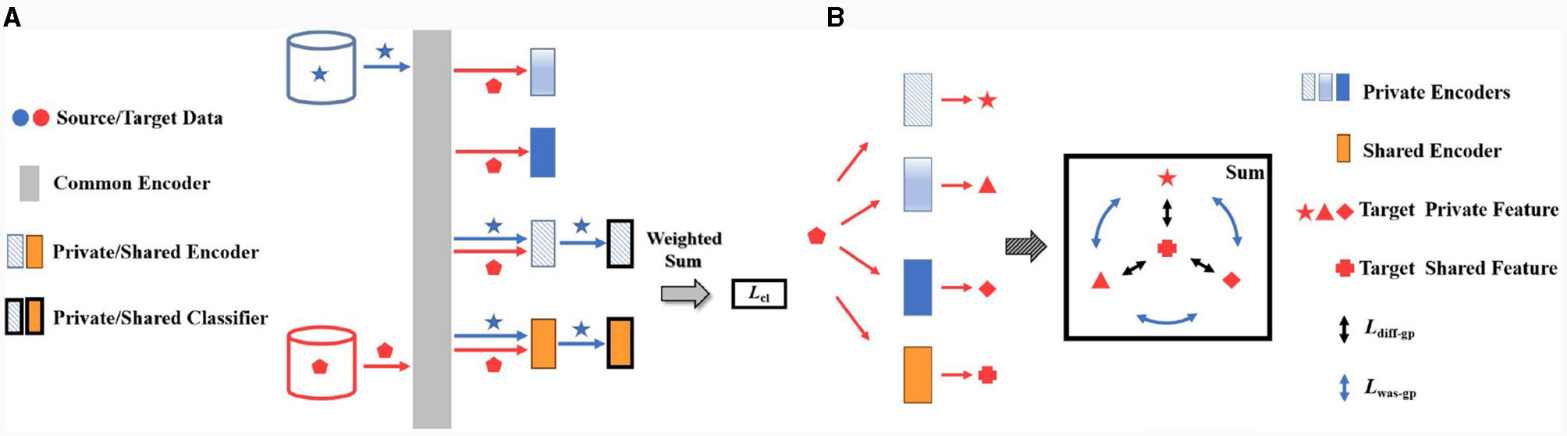
The proposed training strategy in MISNet. **(A)** The proposed two-stream structure, **(B)** loop training strategy.

In order to alleviate the outlier source problem, we propose the two-stream training structure instead of the parallel one, by only inputting the data of current source domain and unlabeled data of target domain during each iteration. The proposed two-stream structure is depicted in Figure 3A. By separating different source domains in the training process, *L*_*was*−*gp*_ is calculated between the current target private feature ${\text{F}}_{TP}^{i}$ and others ${\text{F}}_{TP}^{j}$ (*j*≠*i*), alleviating the effects caused by the other source domains. Similarly, *L*_*diff*−*gp*_ only calculates the distance between the target feature of current private encoder and feature of the shared encoder, avoiding mixing the outlier source domain. As a result, the model errors caused by outlier source domain would be further alleviated.

With the proposed two-stream training structure, we adopt the loop iteration strategy as illustrated in Figure 3B to perform the sequential iterations of all source domains, alleviating domain confusion in loss function and improving the robustness of the network. Here, we refer to a loop of the whole source domains as an epoch comprising *n* iterations, where an iteration corresponds one source domain. Specifically during each iteration, the proposed MISNet successively selects the current source data matrix ${\text{X}}_{S}^{i}$ and its corresponding encoder $E_{P}^{i}$ as the private encoder, then *L*_*mmd*_ measures the marginal distribution between ${\text{F}}_{SP}^{i}$ and ${\text{F}}_{TP}^{i}$, and *L*_*was*−*gp*_ aligns the current target private feature ${\text{F}}_{TP}^{i}$ and others ${\text{F}}_{TP}^{j}$ presented as the blue double arrows in Figure 3B. Meanwhile, *L*_*diff*−*gp*_ aligns the distribution between the current target private feature ${\text{F}}_{TP}^{i}$ and target shared feature **F**_*TS*_ presented as the black double arrows in Figure 3B. During the sequential iterations, the change of source domains in two adjacent iterations will cause slight fluctuations in *L*_*was*−*gp*_ and *L*_*diff*−*gp*_. As the loop iteration progresses, the total loss of the same iteration will decrease between two adjacent loops until it converges.

To make maximum use of the source domain data and ensure that the initial mapping of shared encoder contains sufficient emotional information, we pre-train the common encoder and shared encoder in a subject-dependent emotion classification task. All shuffled source domain data and their labels are used for the training. The common encoder firstly extracts the low-level features, and then the shared encoder is used to capture the deep features. Finally, the shared classifier calculates cross-entropy loss between the output and ground truth labels. In addition, the Adam optimizer is used as the optimizing function, the total epoch is set to 100 and batch size is set to 64. After the pre-training, only the weights of common encoder and shared encoder are saved during the training phase by employing a normally initialized classifier, so that the encoders are initialized to reasonable parameters and the model convergence would be accelerated, which avoids random initialization causing the model to not converge or converge to local optima.

We can also re-examine the network construction from the perspective of loss function design by using the following two criteria:

- *L*_*mmd*_ only reduces the distance between the source and target domains in the private domain instead of using the shared domain.
- The auxiliary losses of *L*_*was*−*gp*_ and *L*_*diff*−*gp*_ use the features of target domain to narrow the corresponding encoder mapping instead of those of source domain.

The purpose of the first criterion is to avoid misleading the direction of domain adaptation when the shared encoder *E*_*S*_ is trained mainly by the classification loss of *L*_*cl*_ which extracts the target domain information containing the individual differences among the source domains.

For the *L*_*was*−*gp*_ loss in the second criterion, if directly using a single source domain ${\text{X}}_{SP}^{i}$ as the input of private encoders and aligning their outputs in each iteration, the wrong gradient of domain information in ${\text{X}}_{S}^{i}$ would be mixed into other encoders ${\text{E}}_{P}^{j}(j\neq i$). Therefore, we use the target private features ${\text{F}}_{TP}^{i}$ and ${\text{F}}_{TP}^{j}$ to align different private domains in *L*_*was*−*gp*_. On the other hand, in the actual iteration process of *L*_*diff*−*gp*_, each private encoder is simultaneously updated by *L*_*cl*_ and *L*_*mmd*_, and different source domain is input in sequence during one loop. When directly adopting the features of source domain as the input will introduce fluctuations in *L*_*diff*−*gp*_, leading to the training collapse. Therefore, we use the features of target domain instead to constrain the domain adaptation direction. After aligning the target private features ${\text{F}}_{TP}^{i}$ and target shared features **F**_*TS*_, the shared classifier *C*_*S*_ could classify the emotion of target domain correctly.

## 4 Experimental settings

This section describes the datasets used for evaluation, EEG data pre-processing and implementation details in the proposed MISNet.

### 4.1 Datasets

We evaluate the proposed network on SEED (Duan et al., 2013; Zheng and Lu, 2015; Liu et al., 2022) and SEED-IV (Zheng et al., 2019), which are public datasets commonly used for EEG emotion recognition. The SEED dataset contains EEG signals from 15 Chinese participants (seven males and eight females). The participants are required to watch 15 Chinese film clips chosen from a pool of materials as stimuli to elicit positive, neutral and negative emotion. Additionally, each film clip contains scenes and audios that is ~4-min long to prevent viewer fatigue. The clip order prevents the continuous display of two clips depicting the same emotion category. Each subject participates in three sessions containing 15 trials, where each session is conducted on a separate day. For feedback purposes, the participants are asked to complete a questionnaire and report their emotional responses immediately after viewing each clip. The EEG signals are recorded by an ESI NeuroScan system at a sampling rate of 1,000 Hz through a 62-electrode cap according to the international 10-20 system. The SEED-IV dataset is similar to the SEED, but it has four emotion categories (happiness, sadness, fear and neutral) and conducts 24 trials per session.

### 4.2 Data pre-processing

For the data pre-processing of EEG signals, the original EEG data was downsampled to 200 Hz and a bandpass filter from 0 to 75 Hz was applied, and a 512-point short-time Fourier transform was used with a non-overlapped Hanning window of 1 s to calculate the frequency domain features. Considering their effectiveness in the EEG emotion recognition task (Yang et al., 2017; Li et al., 2022), the DE features were then computed on the five bands as δ: 1–3 Hz, θ: 4–7 Hz, α: 8–13 Hz, β: 14–30 Hz and γ: 31–50 Hz (Zheng and Lu, 2015). For the gaussian distribution, the DE feature is defined as shown in Equation (18),


(18) $$\begin{array}{l} \text{DE}(X)\text{ = }-\int_{-\infty }^{\infty }\frac{\text{1}}{\sqrt{\text{2}\pi \text{​}\sigma^{\text{2}}}}{\text{e}}^{\frac{{\left(\text{x​}-\text{​}\mu \right)}^{\text{2}}}{\text{2}\sigma^{\text{2}}}}\text{​}\log\text{​}\frac{\text{1}}{\sqrt{\text{2}\pi \text{​}\sigma^{\text{2}}}}{\text{e}}^{-\frac{{\left(\text{x}-\mu \right)}^{\text{2}}}{\text{2}\sigma^{\text{2}}}}dx\text{​}\\ \text{ =}\frac{1}{2}\log\text{ 2}\pi \text{e}\sigma^{\text{2}}, \end{array}$$

where **X** obeys the gaussian distribution *N*(μ, σ^2^), *x* is the element of **X**. Therefore, the 310-dimensional DE features (62 channels multiplying with five frequency bands) were computed, and the features were smoothed with the conventional moving average and linear dynamic system. After the pre-processing steps, each session contains 3,394 samples for the SEED dataset and 822 samples for SEED-IV dataset.

### 4.3 Implementation details

In the proposed MISNet, the common encoder is a 3-layer fully-connected layer with 310-256-128-64 nodes, which extracts the low-level features of source and target domains. Each private encoder and shared encoder are composed of a fully connected layer designed as 64 (input layer)—32 (output layer)—LeaklyRelu activation. Besides, a single fully-connected layer is chosen for each private classifier and shared classifier with a hidden dimension from 32 to the number of emotion categories. Note that there is no batch normalization layer, since we use the gradient penalty guidelines in Equations (9) and (10). The LeakyRelu activation function with a negative slope of 0.01 is used in all hidden layers. In addition, we normalize the data of source and target domains to enhance performance by using the electrode-wise method in Chen et al. (2021a).

For the hyper-parameters in Equation (16), we consider the trade-off among the primary losses of *L*_*cl*_ and *L*_*mmd*_ as well as auxiliary effects of *L*_*was*−*gp*_ and *L*_*diff*−*gp*_, and set $\alpha =\frac{2}{1+e^{-10\times k/\left(nos\times epoch\right)}}-1\left(k=1,2,\cdots \;,nos\times epoch\right)$, where *nos* means number of samples and $\beta =\gamma =\frac{\alpha }{100}$. In our network, we set the learning rate to 0.01 and the batch size to 64. In addition, the Adam optimizer is used as the optimizing function, the total epoch is set to 200, and a cosine annealing schedule is used to determine the learning rate for each epoch. The proposed framework is implemented in PyTorch with version of 1.11 on NVIDIA RTX 1080Ti GPU. The model parameters and computation cost are FLOPs 9.88M and params 154.4K, respectively.

## 5 Experiments and results

In this section, we first test different values of loss weights β and γ to evaluate the effectiveness of the hyper-parameter settings. Next, the ablation experiments are conducted in terms of loss functions, dynamic convergence of network and visualization of mapping features. Then, we compare the proposed network with other competing methods by using the LOSO strategy on the SEED and SEED-IV datasets. Finally, the experiments of adding noise are conducted to demonstrate the robustness of the proposed network.

### 5.1 Hyper-parameter evaluation

**Figure 4:**
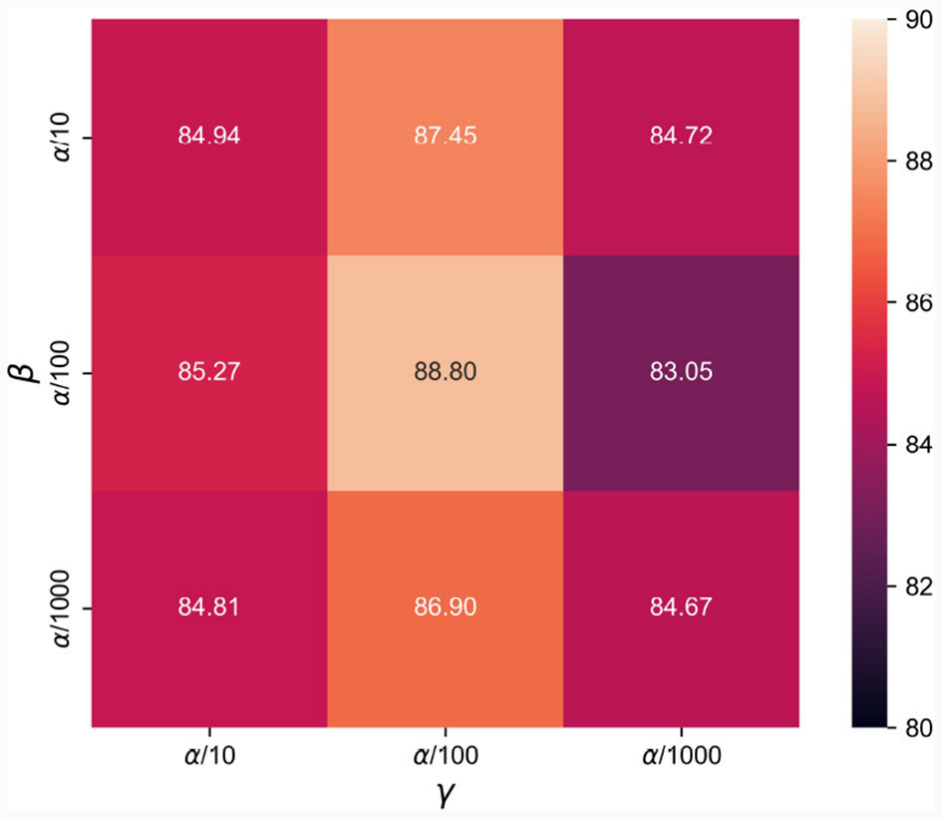
Evaluations of different settings of β and γ in terms of accuracy on the SEED dataset.

In this section, we evaluate the hyper-parameters of β and γ in Equation (16) within a certain range based on previous experience to explore the impact of different settings in terms of accuracy on the SEED dataset. The corresponding results are illustrated in Figure 4. It can be seen from Figure 4 that, both *L*_*was*−*gp*_ and *L*_*diff*−*gp*_ are affected by the selected hyper-parameters of β and γ, with *L*_*diff*−*gp*_ being more sensitive than *L*_*was*−*gp*_. Even with the worst recognition result of 83.05% by setting $\beta =\frac{\alpha }{100}$ and $\gamma =\frac{\alpha }{1000}$, the proposed network still maintains the strong ability of subject independent emotion recognition. Since setting $\beta =\gamma =\frac{\alpha }{100}$ has achieved best recognition performance on SEED dataset, we use this setting to evaluate the effectiveness of network in the following experiments.

### 5.2 Ablation study

To demonstrate the effectiveness of loss functions in MISNet, we evaluate the performance of the ablated network on the SEED and SEED-IV datasets, as shown in Table 1.

**Table 1:** Ablation study of loss functions on SEED and SEED-IV datasets.

**Variants**	**SEED**		**SEED-IV**	
	**Mean**	**Std**.	**Mean**	**Std**.
MISNet	88.80	6.24	74.60	9.30
w/o^*^ gradient penalty	86.52	8.42	67.12	12.65
w/o *L*_*mmd*_	60.47	8.48	56.62	14.22
w/o *L*_*was*−*gp*_	86.72	8.91	70.56	13.15
w/o *L*_*diff*−*gp*_	87.36	10.48	69.50	13.72
*L*_*cl*_ and *L*_*mmd*_	82.71	15.25	67.64	12.26
only *L*_*cl*_	60.20	11.02	54.78	12.35

^*^w/o L_*_ denotes the ablated network trained without the loss function L_*_.

The subject independent recognition performance is evaluated by using the metrics of mean accuracy (Mean) and standard deviation (Std.). Table 1 indicates that all loss functions can improve recognition performance, affording the mean accuracies of 88.80 and 74.60% on the SEED and SEED-IV datasets, respectively. In addition, the proposed MISNet achieves the standard deviation of 6.24 and 9.30% on the SEED and SEED-IV datasets, respectively, showing better inter-subject stability. Discarding *L*_*mmd*_ in our framework leads to a significant performance degradation compared with depriving other loss functions, proving its importance in domain adaptation. And thus, the higher weight should be assigned to the loss of *L*_*mmd*_ than those of *L*_*was*−*gp*_ and *L*_*diff*−*gp*_. Furthermore, removing *L*_*was*−*gp*_ and *L*_*diff*−*gp*_ simultaneously will damage the domain adaptability more than removing any of them individually, since they control the relationship between private features and shared features jointly. In addition, the gradient penalty of two auxiliary loss functions *L*_*was*−*gp*_ and *L*_*diff*−*gp*_ allows stable convergence of the private and shared domains, which is reflected in the improvement of average accuracy and decrease of standard deviation.

**Figure 5:**
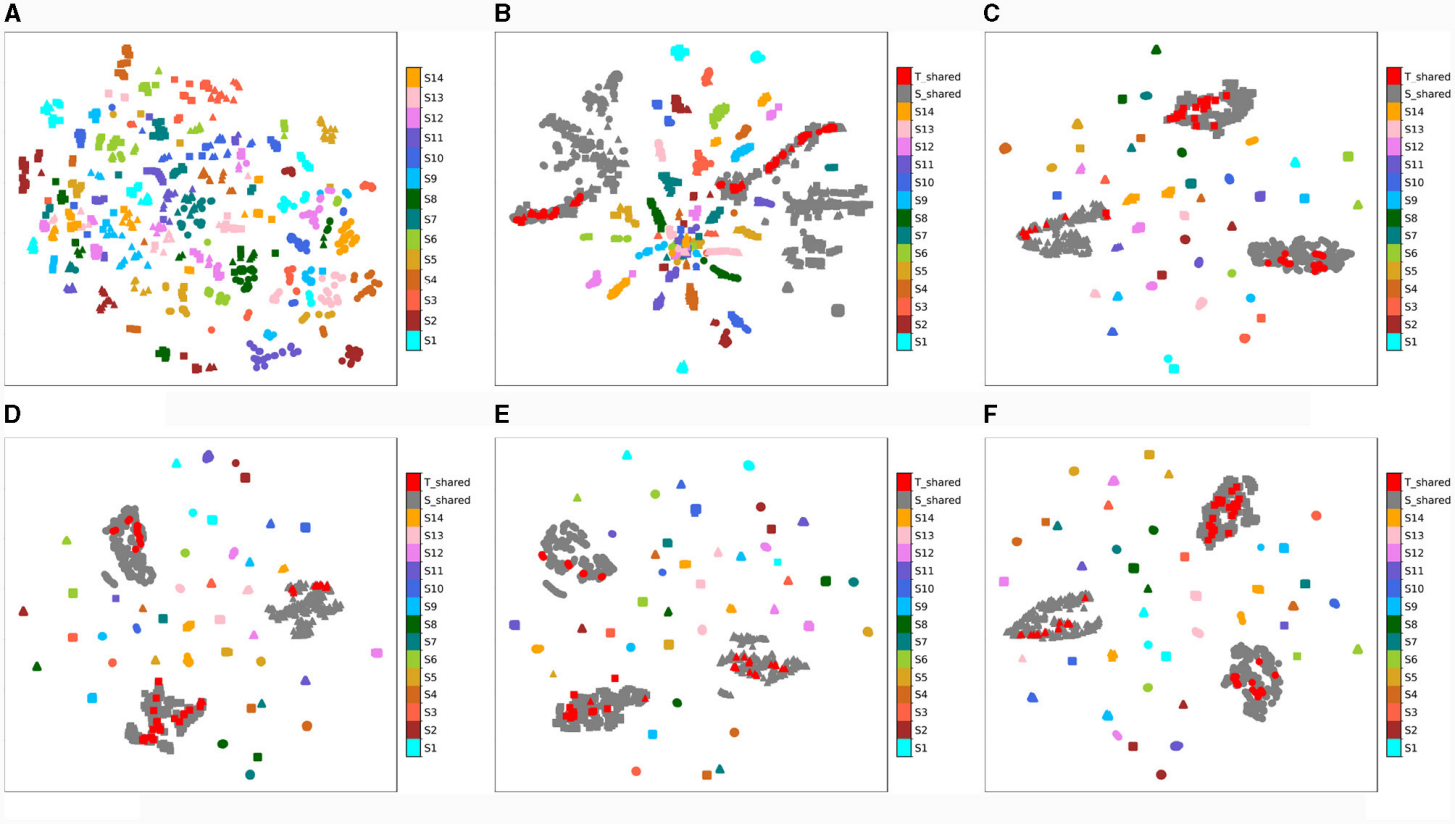
Training process of the proposed MISNet with t-sne mapping. Red color denotes the target shared features and gray color represents the source shared features, while other colors symbolize the source private features. Different shapes denote the different emotion categories: •, ▴, ■ represents positive, neutral and negative emotions, respectively. **(A)** the original distributions of the DE feature projection of different source domains, **(B)** the initialization effect of subject-dependent pre-training, **(C–F)** the training process of MISNet.

One of the primary goals of the proposed network is to align the distributions of private and shared domains to alleviate the negative transfer in domain adaptation caused by individual differences in EEG signals. Next, we evaluate the convergence process of proposed MISNet and visualize the domain mapping of the private and shared domains in a two-dimensional way by using the t-distributed stochastic neighbor embedding (t-sne). The t-sne method is employed to evaluate the similarity between feature representations during the training process, meaning the closer points have a higher similarity in real space. The similarity between source and target domains affects the domain adaptability of network, while the distinction among the emotion categories reflects its emotion discrimination. The dynamic convergence process of the proposed MISNet is shown in Figure 5.

It can be depicted from Figure 5A that the original distributions of the DE feature projection of different source domains are chaotic and irregular, due to the individual differences of EEG signals. After the designed subject-dependent pre-training strategy, the different emotional categories of source shared domain represented by different gray shapes have been distinguished to some extent, which means the shared encoder has a fundamental emotion classification ability, as illustrated in Figure 5B. Figures 5C–F reveal that as the convergence process progresses, the private domain of each source domain (represented by different colors) is clustered based on their emotion category (represented by different shapes), indicating a gradual improvement of emotional discrimination. In addition, the cluster center of each domain is distributed near the middle of the figure, indicating that all cluster centers are aligned among the private domains. As the convergence process progresses, the space of private domains gradually shrinks, indicating that the model is eliminating the interference of spacing among private domains. Furthermore, it can also be found from Figure 5F that, when the model has converged, the source private domains exhibit different emotional distribution patterns, this is because we align the domain centers instead of aligning the distribution of private features in *L*_*was*−*gp*_. Additionally, the target shared features (represented by red) are almost always within the source shared features with corresponding emotion categories (represented by gray), indicating that the shared encoder can effectively capture the shared emotion information. After the final optimization, the center of the shared domains roughly coincides with that of the private domains, and the boundary of shared domains is close to that of maximum private domain. It can be concluded that the convergence process of MISNet is identical to the anticipated and has a strong domain adaptability for subject independent EEG emotion recognition.

### 5.3 Comparisons with competing methods

In this section, we compare the proposed MISNet with several competing methods on the SEED and SEED-IV datasets. Table 2 shows the comparison results in terms of the mean classification accuracy and standard deviation with competing methods. Here the results of MS-MDA (Chen et al., 2021a) were obtained by using the LOSO strategy with the open source codes.^1^

**Table 2:** Comparison results of the proposed MISNet with competing methods on the SEED and SEED-IV datasets.

**Methods**	**SEED**		**SEED-IV**	
	**Mean**	**Std**.	**Mean**	**Std**.
SVM (Zheng and Lu, 2016)	56.7	4.6	−	−
WGANDA (Luo et al., 2018)	87.1	7.1	−	−
DAN (Li H. et al., 2018)	83.8	8.6	−	−
DResNET (Ma et al., 2019)	85.3	8.0	−	−
PPDA (Zhao et al., 2021)	86.7	7.1	−	−
MS-MDA (Chen et al., 2021a)	82.9	6.4	62.8	10.9
MEER-NET (Chen et al., 2021b)	87.1	2.0	71.0	12.1
wMADA-β (Luo and Lu, 2021)	**89.3**	4.0	−	−
MWACN (Zhu et al., 2022)	87.6	4.0	**74.4**	−
MISDA (Gong et al., 2022)	**88.1**	9.5	**73.8**	11.9
**MISNet (Ours)**	**88.8**	6.2	**74.6**	9.3

The bold font in the table represents the top three in the results.

It can be seen from Table 2 that the domain adaptation-based methods significantly improve the recognition performance compared with directly using SVM in the subject independent experiments. Furthermore, most domain adaptation methods using multi-source (Chen et al., 2021a,b; Luo and Lu, 2021; Gong et al., 2022; Zhu et al., 2022) can attain better performance than those without multi-source (Li H. et al., 2018; Luo et al., 2018; Ma et al., 2019), which indicates the importance of considering individual differences inside the source domains. Specifically, the proposed MISNet outperforms most of the competing methods, achieving a mean accuracy of 88.8 and 74.6% on the SEED and SEED-IV datasets, respectively. This is attributed to the designed loss functions *L*_*was*−*gp*_ and *L*_*diff*−*gp*_, which enhance the domain adaptability of the network. The evaluation index of standard deviation means the stability performance of networks across subjects in the dataset. Compared with the typical existing methods on two benchmark datasets of SEED and SEED-IV, the proposed MISNet demonstrates the competitive ability overcome the individual differences. Although a small gap exists compared to wMADA-β (Luo and Lu, 2021), the proposed method has simpler parameter tuning process during the training phase compared to wMADA-β (Luo and Lu, 2021).

In order to prove the generality of the proposed model, we compare it with the competitive method of MS-MDA (Chen et al., 2021a) by using the indicators of F1 score, sensitivity and specificity in Table 3. In the experiment, all folded performance was used to estimate the indicators of F1 score, sensitivity and specificity under the LOSO strategy. It can be seen from Table 3 that, the proposed MISNet has higher generalization ability on all three aspects than MS-MDA (Chen et al., 2021a) and performs stably in all subjects.

**Table 3:** Comparison generality with competing method on the SEED and SEED-IV datasets.

**Variants**	**SEED**			**SEED-IV**		
	**F1-score**	**Sensitivity**	**Specificity**	**F1-score**	**Sensitivity**	**Specificity**
MS-MDA (Chen et al., 2021a)	79.8 ± 15.1	80.1 ± 15.2	90.3 ± 7.4	42.8 ± 15.4	44.9 ± 14.8	81.8 ± 5.2
MISNet (Ours)	88.7 ± 7.3	88.8 ± 7.2	94.5 ± 3.1	73.2 ± 13.3	74.1 ± 11.9	92.3 ± 3.9

**Figure 6:**
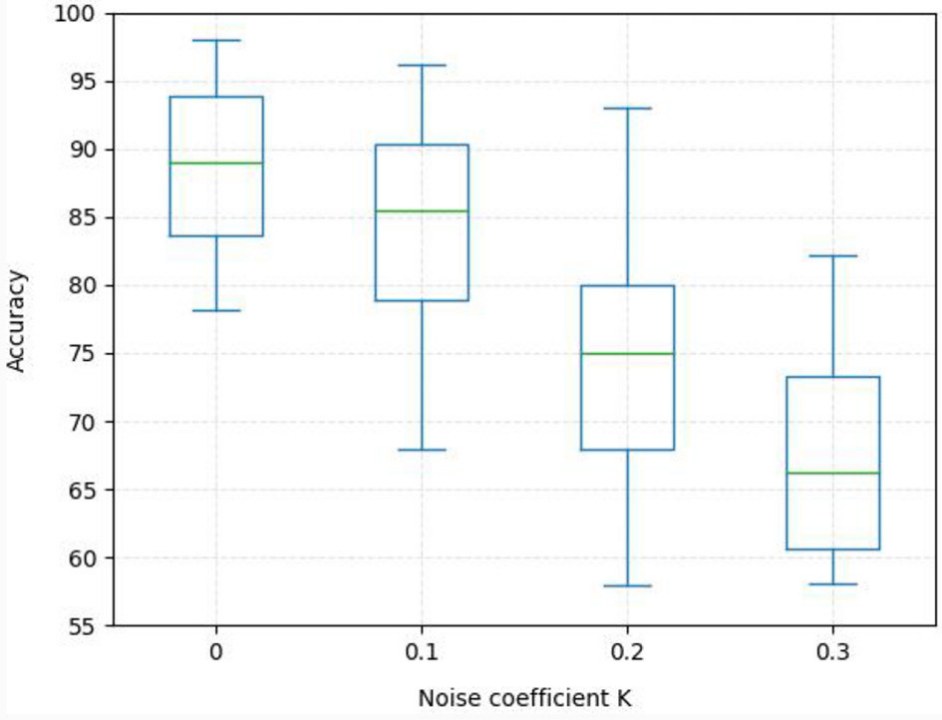
The robustness of MISNet to noise on the SEED dataset.

To further demonstrate the robustness of the proposed MISNet network, we evaluate the performance of the network on the SEED dataset by adding gaussian noise in the test data, as shown in Equation (19),


(19) $$\begin{array}{l} X_{T}^{N}=X_{T}+KN, \end{array}$$

where **X**_*T*_ are the target matrices, *N* denotes the gaussian noise which obeys the gaussian distribution *N*(0, 1) and *K* is the noise coefficient. In the experiment, we verify the robustness of the proposed network to noise by gradually increasing the noise coefficient, as shown in Figure 6. It can be seen from Figure 6 that, with the noise coefficient *K* increasing from 0 to 0.3, that is, the signal-to-noise ratio gradually decreases, the recognition accuracy of the model gradually decreases inevitably, and the recognition variance increases. When the signal-to-noise ratio is relatively large, that is, when *K* is equal to 0.1 and 0.2, the mean accuracy of the proposed model still remains above 75%, indicating that it has a strong tolerance for noise.

**Figure 7:**
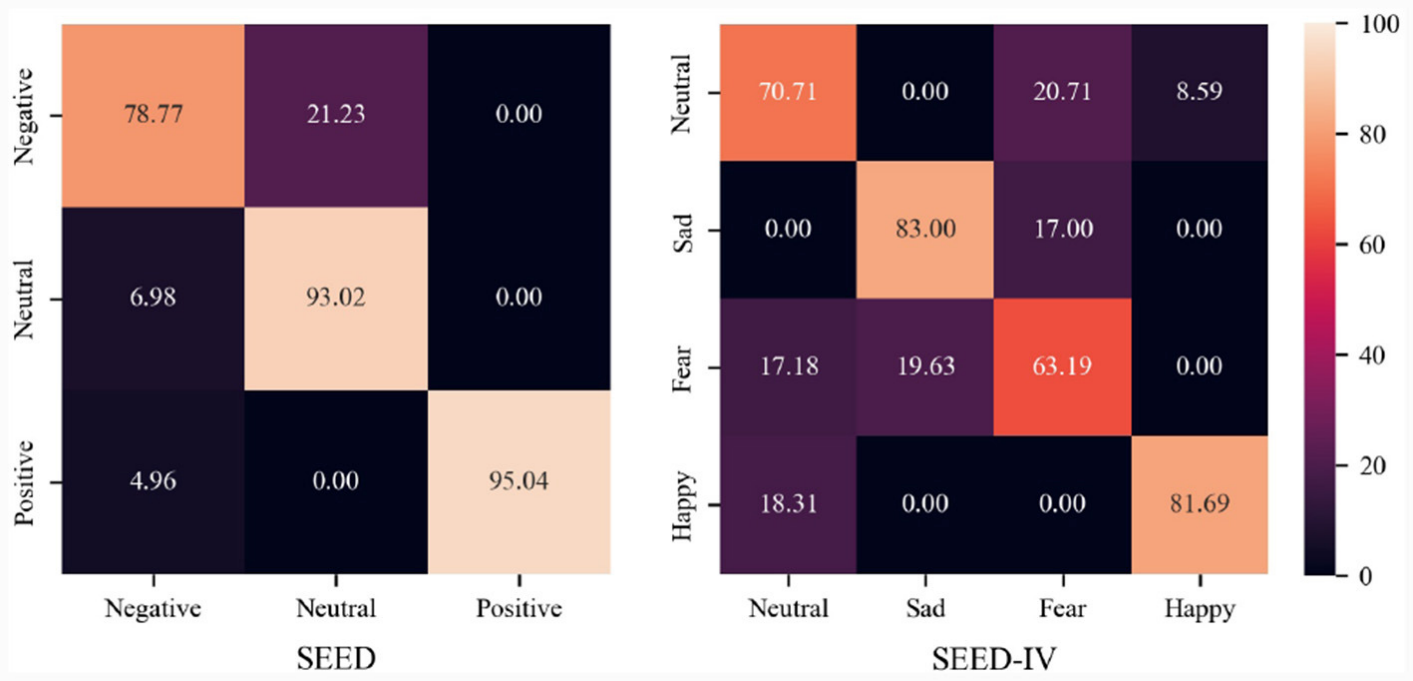
Confusion matrices of predictions on SEED and SEED-IV dataset.

In order to show the recognition ability of the proposed MISNet among different emotion categories, Figure 7 shows the confusion matrix on the SEED and SEED-IV datasets. It can be seen from Figure 7 that on the SEED dataset, the MISNet achieves the recognition accuracies of 78.77, 93.02, and 95.03%, respectively on the emotion categories of negative, neutral and positive, demonstrating strong discriminative capability across emotions. And the results on the SEED-IV dataset infer that our framework has decent accuracies for the emotion categories of neutral, sad and happy. While for the category of fear, the proposed MISNet confuses it with sad because these two emotions are relatively similar on EEG signals.

## 6 Conclusion

Although the EEG signals have the advantage of spontaneous and non-subjective characteristic in emotion recognition filed, they still have several limitations, including the individual difference and noisy labeling issues. In this paper, the main challenge we aim to address is the domain shift problem caused by the non-stationary of EEG signals and the individual differences among users.

For the purpose of alleviating the domain shift problem, we propose to consider the individual differences and group commonalities simultaneously, improving the domain adaptation ability of the model. In the proposed MISNet, the decoupling network structure is designed to extract the private domain features and shared domain features of each domain data. In order to constrain overall optimization direction, the classification loss function and domain adaptation loss functions are adopted. In addition, we analyze the convergence process of network to design the auxiliary loss functions of *L*_*was*−*gp*_ and *L*_*diff*−*gp*_ in order to align the different domain centers. A pre-training strategy is also used to enhance model stability and ensure that the initial mapping of shared encoder contains sufficient emotional information. Furthermore, the convergence process of the proposed network is dynamically displayed through t-sne mapping. The results on the SEED and SEED-IV datasets demonstrate the effectiveness of our proposed MISNet frameworks.

Since the proposed MISNet needs the unlabeled data of target domain to obtain domain information, it is available for the offline situations in real life, and achieves high-quality emotional awareness by decoupling personality and common emotional characteristics. Our future work will focus on disentangling the domain information from EEG data with a reasonable explanation, thereby constructing a more robust network.

## Data availability statement

Publicly available datasets were analyzed in this study. This data can be found here: https://bcmi.sjtu.edu.cn/home/seed/index.html; https://bcmi.sjtu.edu.cn/home/seed-iv/index.html.

## Ethics statement

The studies involving humans were approved by Ethics and Morality Committee of Communication University of China. The studies were conducted in accordance with the local legislation and institutional requirements. Written informed consent for participation was not required from the participants or the participants' legal guardians/next of kin in accordance with the national legislation and institutional requirements.

## Author contributions

MG: Conceptualization, Data curation, Writing—original draft, Formal analysis, Methodology. WZ: Conceptualization, Writing—review & editing, Formal analysis, Funding acquisition. LY: Investigation, Writing—review & editing, Validation, Visualization. QZ: Funding acquisition, Writing—review & editing, Supervision, Project administration.

## Footnotes

^1^ https://github.com/VoiceBeer/MS-MDA
